# Effect of remote ischemic post-conditioning on systemic inflammatory response and survival rate in lipopolysaccharide-induced systemic inflammation model

**DOI:** 10.1186/1476-9255-11-16

**Published:** 2014-05-21

**Authors:** Yun-Hee Kim, Dae-Wui Yoon, Je-Hyeong Kim, Jeoung-Hyuk Lee, Choon-Hak Lim

**Affiliations:** 1 Department of Anaesthesiology and Pain Medicine, Korea University College of Medicine, Ansan, Republic of Korea; 2 Division of Pulmonary, Sleep and Critical Care Medicine, Department of Internal Medicine, Korea University College of Medicine, Ansan, Republic of Korea; 3 Department of Anaesthesiology and Pain Medicine, Dongguk University Ilsan Hospital, Ilsan, Republic of Korea; 4 Department of Anaesthesiology and Pain Medicine, Korea University College of Medicine, Seoul, Republic of Korea

**Keywords:** Remote ischemic preconditioning, Remote ischemic postconditioning, Systemic inflammation

## Abstract

**Background:**

Remote ischemic preconditioning (RIPC) and postconditioning (RpostC) have protective effects on ischemia and reperfusion injury. The effects have been reported to activate heme oxygenase-1 (HO-1) and attenuate nuclear factor kappa B (NF-κB) and subsequently reduce systemic inflammation. Ischemic preconditioning prevented inflammatory responses by modulating HO-1 expression in endotoxic shock model. Therefore, we investigated whether RpostC could have protective effects on lipopolysaccharide (LPS)-induced systemic inflammation.

**Methods:**

The LPS-induced sepsis mice received LPS (20 mg/kg) intraperitoneally. Remote ischemic conditioning was induced with three 10-min ischemia/10-min reperfusion cycles of the right hind limbs using tourniquet before LPS injection (RIPC) or after LPS injection (RpostC). The effects of RIPC and RpostC were examined for the survival rate, serum cytokines, NF-κB, HO-1 and liver pathology in the LPS injected mice.

**Results:**

Survival rate within 120 hours significantly increased in the LPS injected and remote ischemic conditioned mice than in LPS only injected mice (60-65% vs 5%, respectively, p < 0.01). Tumor necrosis factor-alpha (TNF-α), interleukin-1 beta (IL-1β) and interleukin-6 (IL-6) increased markedly in the LPS only injected mice, however, remote ischemic conditioning suppressed the changes (p < 0.05). Interleukin-10 (IL-10) level was significantly higher in the LPS injected and RpostC treated mice than in the LPS only injected mice (*p* = 0.014). NF-κB activation was significantly attenuated (p < 0.05) and HO-1 levels were substantially higher in the LPS injected and remote ischemic conditioned mice than in the LPS only injected mice. Neutrophil infiltration was significantly attenuated in the LPS injected and remote ischemic conditioned mice than in the only LPS injected mice (p < 0.05).

**Conclusions:**

RpostC attenuated inflammatory responses and improved survival outcomes of mice with LPS-induced systemic inflammation. The mechanism may be caused by modifying NF-κB mediated expression of cytokines.

## Background

Severe sepsis is a life-threatening clinical disease induced by infection or other various causes and is characterized by systemic inflammation and multiple organ injury. In spite of development of new therapies for sepsis, mortality rate is approximately 30% in people with severe sepsis and up to 45% of those in septic shock [1]. Therefore, new treatment methods for sepsis need to be discovered and developed.

The liver plays an active role in the inflammatory response to endotoxemia and sepsis by producing acute phase proteins and proinflammatory cytokines [2,3]. Nuclear factor kappa B (NF-κB) is activated in an early step of pathogenesis of organ injury in sepsis [4,5]. However, NF-κB activation is known to be inhibited by heme oxygenase-1 (HO-1) activation [6,7]. The expression of HO-1 can be induced by ischemic preconditioning [8,9], ischemic postconditioning [10,11] or remote ischemic predonditioning (RIPC) [12-14]. Remote ischemic preconditioning (RIPC) or postconditioning (RpostC), achieved with repeated brief periods of ischemia and reperfusion (I/R) of one organ before or after prolonged ischemic period has been reported to protect distant organs against ischemic injury [15]. The effects are associated with down-regulation of key steps leading to cell death and systemic inflammatory responses [10,15-17]. Indeed, intestinal ischemic preconditioning prevented inflammatory responses by modulating intestinal HO-1 expression in endotoxic shock model [18]. Furthermore, Wen et al. [19] reported upregulation of HO-1 by chemical inducers prevented lipopolysaccharide (LPS)-induced acute hepatic injury.

Therefore, we hypothesized that RpostC may attenuate LPS-induced systemic inflammatory responses by inducing HO-1 expression and attenuating NF-κB activation. There has been little work, however, examining the effects of RpostC in the setting of LPS-induced sepsis model. We investigated whether RpostC could improve survival rate and suppress LPS-induced pro-inflammatory cytokines, NF-κB activation, and hepatic inflammation. Also, we assessed if RpostC might upregulate HO-1, leading to the attenuation of LPS-induced systemic inflammation.

## Methods

### Animals

Six-week-old male BALB/c mice (Hanlim Co. Ltd., Hwasung, South Korea) were used in this study. All animals were maintained in a constant-temperature (24 ± 2°C) room with 12-hour cycles of light and dark. The mice had ad libitum access to food and water.

The LPS-induced sepsis model was made by injecting 20 mg/kg of LPS (E, Coli O127: B8; Sigma, St. Louis, MO, USA) in 0.4 ml physiologic saline intraperitoneally (i.p.). Remote conditioning was induced with three 10-min ischemia/10-min reperfusion cycles of the right hind limbs using tourniquet.

All experimental procedures involving animals were carried out in accordance with the NIH guide for the Care and Use of Laboratory Animals issued by the Korea University School of Medicine. The study was approved by the Ethical Committee on Animal Research of the Korea University College of Medicine.

**Figure 1:**
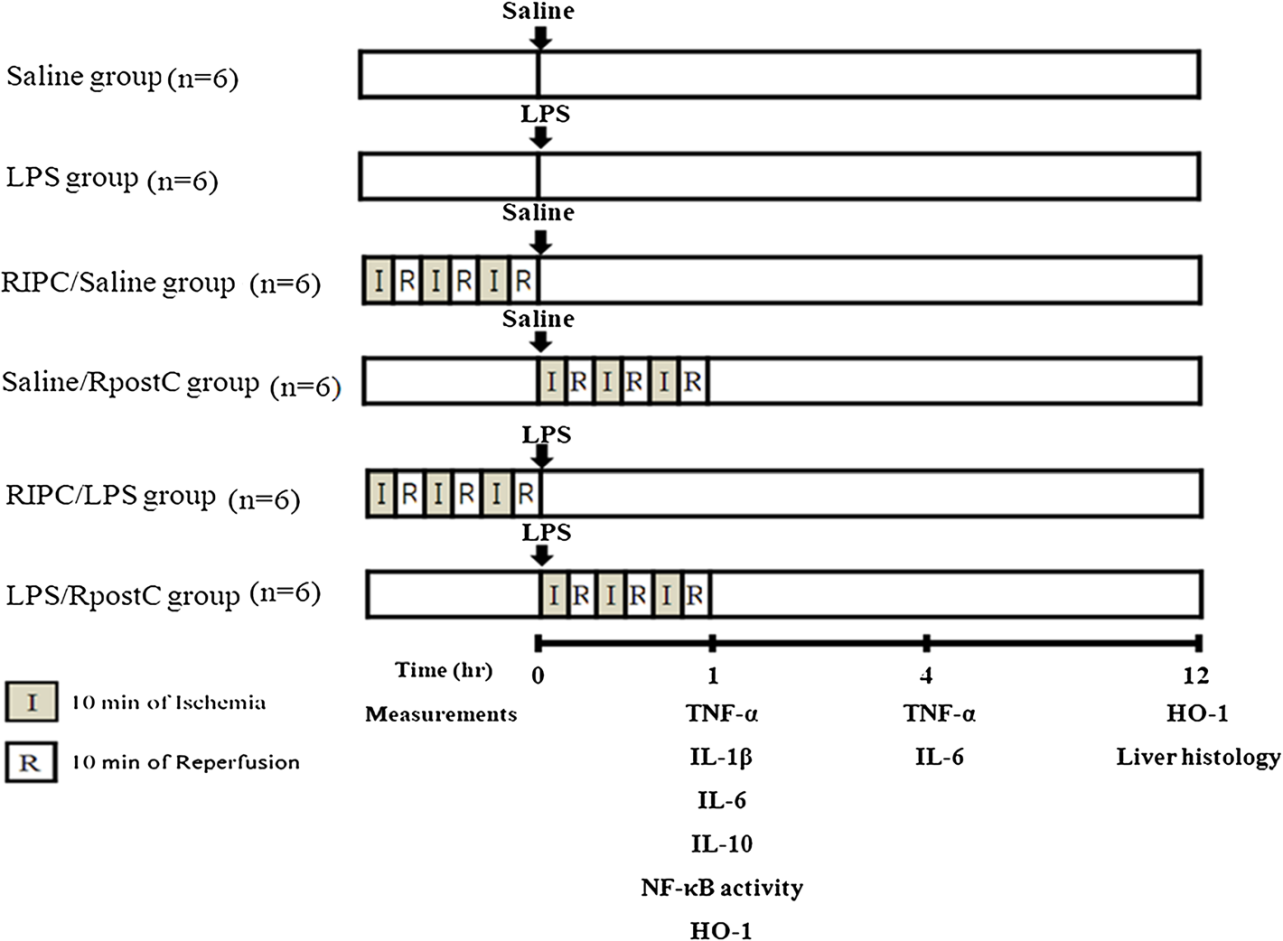
**Experimental protocol.** Mice were randomly divided into six experimental groups (n = 6, each group) according to each target measurements: (1) Saline group, received an injection of saline (intraperitoneally: i.p.); (2) LPS group, received an injection of LPS (20 mg/kg, i.p.); (3) RIPC/Saline group, subjected to remote conditioning, followed immediately by an i.p. injections of saline; (4) Saline/RpostC group, received an injection of saline i.p., followed immediately by remote conditioning; (5) RIPC/LPS group, was subjected to remote conditioning, followed immediately by an injection of LPS (20 mg/kg, i.p.); (6) LPS/RpostC group, received an injection of LPS (20 mg/kg, i.p.), followed immediately by remote conditioning. Remote conditioning was induced with three 10-min ischemia/10-min reperfusion cycles of the right hind limbs using tourniquet.

Mice were randomly divided into six experimental groups: (1) Saline group, received an injection of saline i.p.; (2) LPS group, received an injection of LPS (20 mg/kg, i.p.); (3) RIPC/Saline group, subjected to remote conditioning, followed immediately by an i.p. injections of saline; (4) Saline/RpostC group, received an injection of saline i.p., followed immediately by remote conditioning; (5) RIPC/LPS group, subjected to remote conditioning, followed immediately by an injection of LPS (20 mg/kg, i.p.); (6) LPS/RpostC group, received an injection of LPS (20 mg/kg, i.p.), followed immediately by remote conditioning (Figure 1).

### Protocol 1: Survival rate

Survival rate was measured in the LPS group (n = 40), RIPC/LPS group (n = 20), and LPS/RpostC (n = 20) groups, every 8 hours for 120 hours. After 5 days, all living mice were euthanatized with intraperitoneal injection of thiopental sodium (50 mg/kg).

### Protocol 2: cytokines, NF-κB, HO-1 and liver pathology

Mice were randomly divided into six experimental groups (n = 6, each group) according to each target measurements.

Serum pro-inflammatory cytokines (TNF-α, IL-6) levels were measured at 1 hour and 4 hours after LPS (or saline) injection and another pro-inflammatory cytokine (IL-1β) and anti-inflammatory cytokine (IL-10) levels were at 1 hour after LPS injection (or saline) in each group. Blood was obtained directly from the heart. Cytokines were determined in the serum using commercial enzyme linked immunosorbent assay (ELISA) assay kits (R&D Systems, Minneapolis, USA). After sampling *via* cardiac puncture, serum was collected by centrifuging the clotted blood at 4,000 × g, 4°C, 10 min. Detection ranges of TNF-α, IL-1β, and IL-6 are 0.36-7.21, 0.46-4.80, and 1.3-1.8 pg/ml, respectively. The minimal detectable doses of TNF-α, IL-1β, IL-6, and IL-10 are less than 1.88, 2.31, 1.6, and 4.0 pg/ml, respectively.

The NF-κB activity was measured at 1 hour after LPS (or saline) injection in liver tissue homogenate. Nuclear protein was extracted in the liver tissue using a commercial kit according to the instruction manual (Nuclear Extract Kit, Active Motif, California, USA). Nuclear protein concentration was determined using BCA protein assay kit (Pierce, Rockford, IL, USA). Five μg of nuclear protein from liver tissues was used to assess NF-kB activation using the NF-κB p65 assay kit (Trans^AM^ p65, Active Motif, USA) according to the manufacturer’s instruction.

HO-1 was measured in the supernatant of homogenized liver extracts at 1 hour and 12 hours after LPS (or saline) injection using the mouse HO-1 ELISA kit according to the manufacturer’s instruction (Cusabio Bio-Tech, Wuhan, China). One hundred mg of liver tissue was rinsed with phosphate buffered saline (PBS) and homogenized in 1 ml of PBS. The homogenate was stored overnight at -20°C. After two freeze-thaw cycles were performed to break the cell membranes, the homogenates were centrifuged for 5 min at 5000 × g, 4°C. The supernatant was used for HO-1 assay with 1,500-fold dilution with sample diluents. Detection range of the assay is 31.25 ng/ml - 2000 ng/ml, and the minimal detectable dose of HO-1 is less than 7.8 pg/ml.

Liver tissues were collected at 12 hours after LPS injection. The right lobes of the liver were fixed with 10% neutral buffered saline for 24 hours in room temperature, embedded in paraffin, and cut into 4 μm sections. Tissue sections were then stained with anti-Ly-6G stain for neutrophil count. Neutrophil accumulation was quantified by a researcher who was blinded towards experiment protocol. Brown color-stained PMNs (polymorphonuclear neutrophils) were counted in 16 random high-power fields (400x), and the averaged values were reported.

### Statistical analysis

All data were expressed as the mean ± standard error of the mean (SEM). Survival data were analyzed using Kaplan-Meier log-rank test. For detection of significant differences between groups a Kruskal-Wallis test was used. A post hoc analysis was then performed with pairwise Mann–Whitney U tests. For all tests, a p-value less than 0.05 was considered statistically significant. All statistical analyses were performed using SPSS, version 12*.*0 (SPSS^®^, Chicago, Illinois, USA).

## Results

### Survival rate

The number of survivors was 2/40, 13/20, and 12/20 in the LPS, RIPC/LPS, and LPS/RpostC groups, respectively.

**Figure 2:**
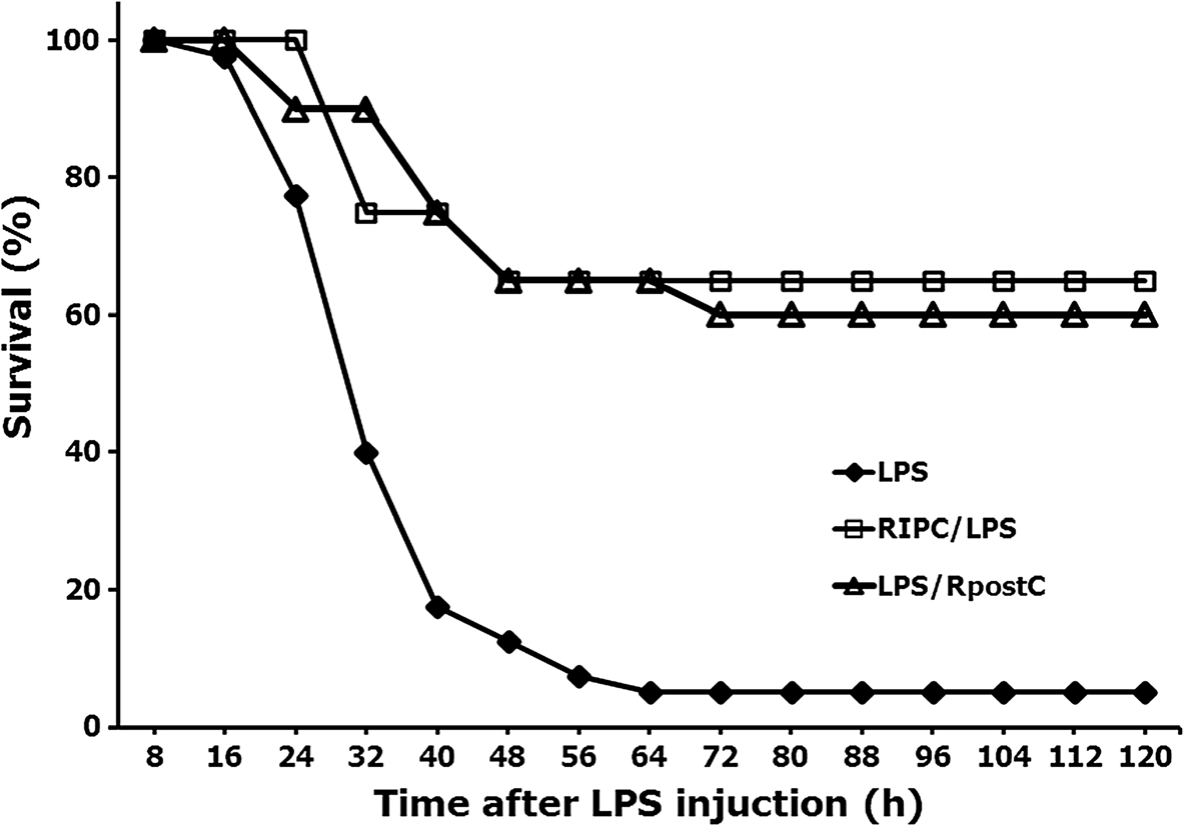
**Survival rate.** The survival rate of RIPC/LPS (n = 20) and LPS/RpostC (n = 20) groups were significantly greater than that of LPS group (n = 40) (p < 0.001, p < 0.01, respectively). There was no significant difference between the RIPC/LPS and LPS/RpostC groups. LPS group, received an injection of LPS (20 mg/kg, i.p.); RIPC/LPS group, was subjected to remote conditioning, followed immediately by an injection of LPS (20 mg/kg, i.p.); LPS/RpostC group, received an injection of LPS (20 mg/kg, i.p.), followed immediately by remote conditioning. RIPC: remote ischemic preconditioning, RpostC: remote ischemic postconditioning.

The survival rate of RIPC/LPS and LPS/RpostC groups were significantly greater than that of LPS group (p < 0.001, p < 0.01, respectively) (Figure 2). There was no significant difference between the RIPC/LPS and LPS/RpostC groups.

### Cytokines

**Figure 3:**
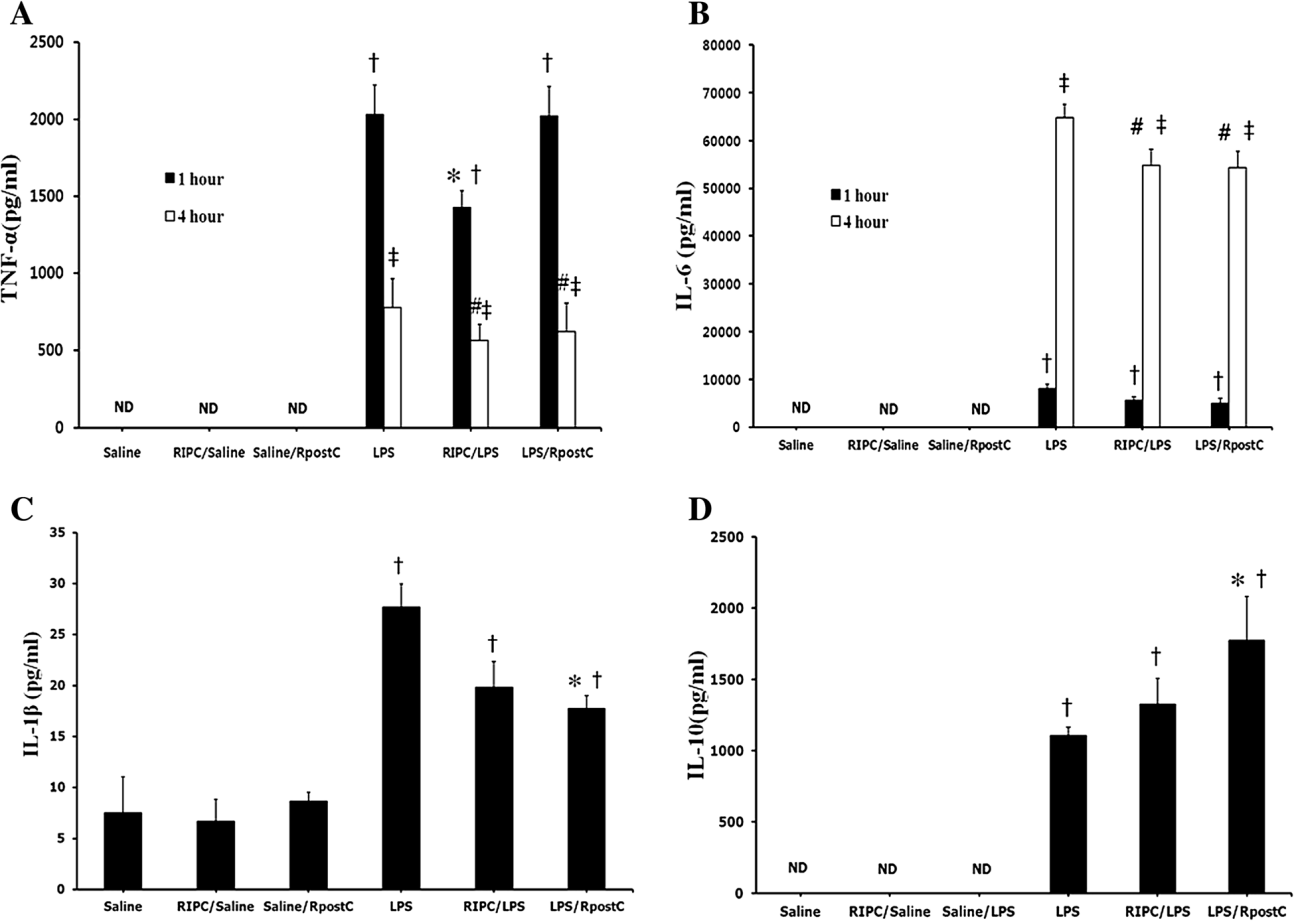
**Effects of RIPC and RpostC on levels of TNF-α, IL-6, IL-1β and IL-10.** The concentration of TNF-α **(A)** and IL-6 **(B)** levels were measured at 1 hour and 4 hours after LPS (or saline) injection. IL-1β **(C)** and IL-10 **(D)** levels were at 1 hour after LPS injection (or saline) in each group. Mice were treated with saline (Saline group), RIPC plus saline (RIPC/Saline group), saline plus RpostC (Saline/RpostC group), LPS (20 mg/kg, i.p.; LPS group), RIPC plus LPS (RIPC/LPS group), and LPS plus RpostC (LPS/RpostC group). i.p.: intraperitoneal. RIPC: remote ischemic preconditioning, RpostC: remote ischemic postconditioning. Data are expressed as mean ± SEM (n = 6 in each group). † *P* < 0.05 versus Saline, RIPC/Saline, Saline/ RpostC at 1 h after LPS injection or saline injection. *P < 0.05 versus LPS group at 1 h after LPS injection or saline injection. ‡ *P* < 0.05 versus Saline, RIPC/Saline, Saline/ RpostC at 4 h after LPS injection or saline injection. # *P* < 0.05 versus LPS group at 4 h after LPS injection or saline injection. ND, not detectable.

The TNF-α level was significantly lower in the RIPC/LPS group than in the LPS group at 1 hour after LPS administration (p = 0.009) and was significantly lower in the RIPC/LPS and LPS/RpostC group than in the LPS group at 4 hours after LPS administration (*p* = 0.006 and *p* = 0.006, respectively) (Figure 3A).

IL-6 levels in the RIPC/LPS and LPS/RpostC group revealed a significant decrease compared with those in LPS group at 4 hours (*p* = 0.019, *p* = 0.03, respectively) (Figure 3B).

The IL-1β level was significantly lower in the LPS/RpostC group than in the LPS group (*p* = 0.013) (Figure 3C).

Serum IL-10 level was significantly higher in the RpostC/LPS group compared with LPS group (*p* = 0.014) (Figure 3D).

### NF-κB DNA binding activity

**Figure 4:**
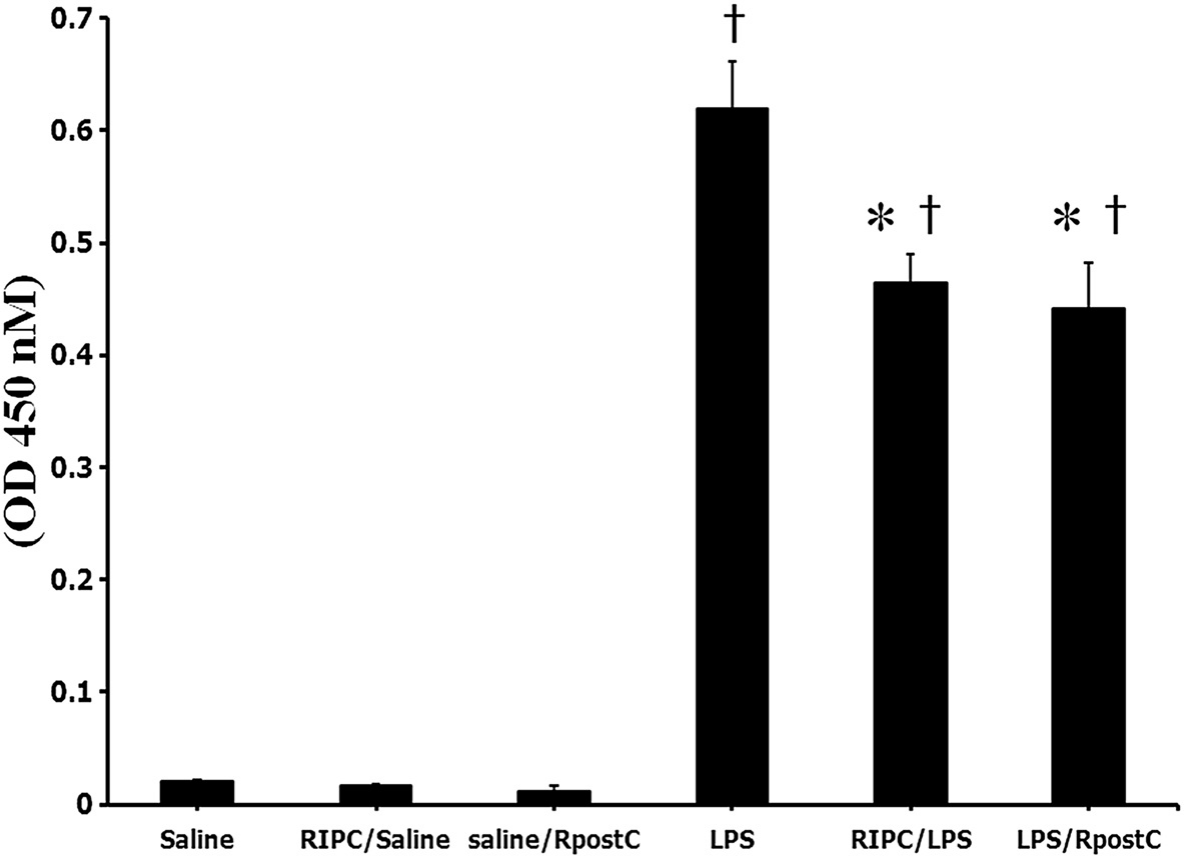
**NF-κB DNA binding activity.** The NF-κB activity was measured at 1 hour after LPS (or saline) injection in liver tissue homogenate. Mice were treated with saline (Saline group), RIPC plus saline (RIPC/Saline group), saline plus RpostC (Saline/RpostC group), LPS (20 mg/kg, i.p.; LPS group), RIPC plus LPS (RIPC/LPS group), and LPS plus RpostC (LPS/RpostC group). i.p.: intraperitoneal. RIPC: remote ischemic preconditioning, RpostC: remote ischemic postconditioning. Data are expressed as mean ± SEM (n = 6 in each group). *P < 0.05 versus LPS group. † *P* < 0.05 versus Saline, RIPC/Saline, Saline/RpostC.

NF-κB DNA binding activity in the RIPC/LPS and LPS/RpostC group revealed a significant decrease when compared with those in the LPS group (*p* = 0.021 and *p* = 0.006, respectively) (Figure 4).

### HO-1 activity

**Figure 5:**
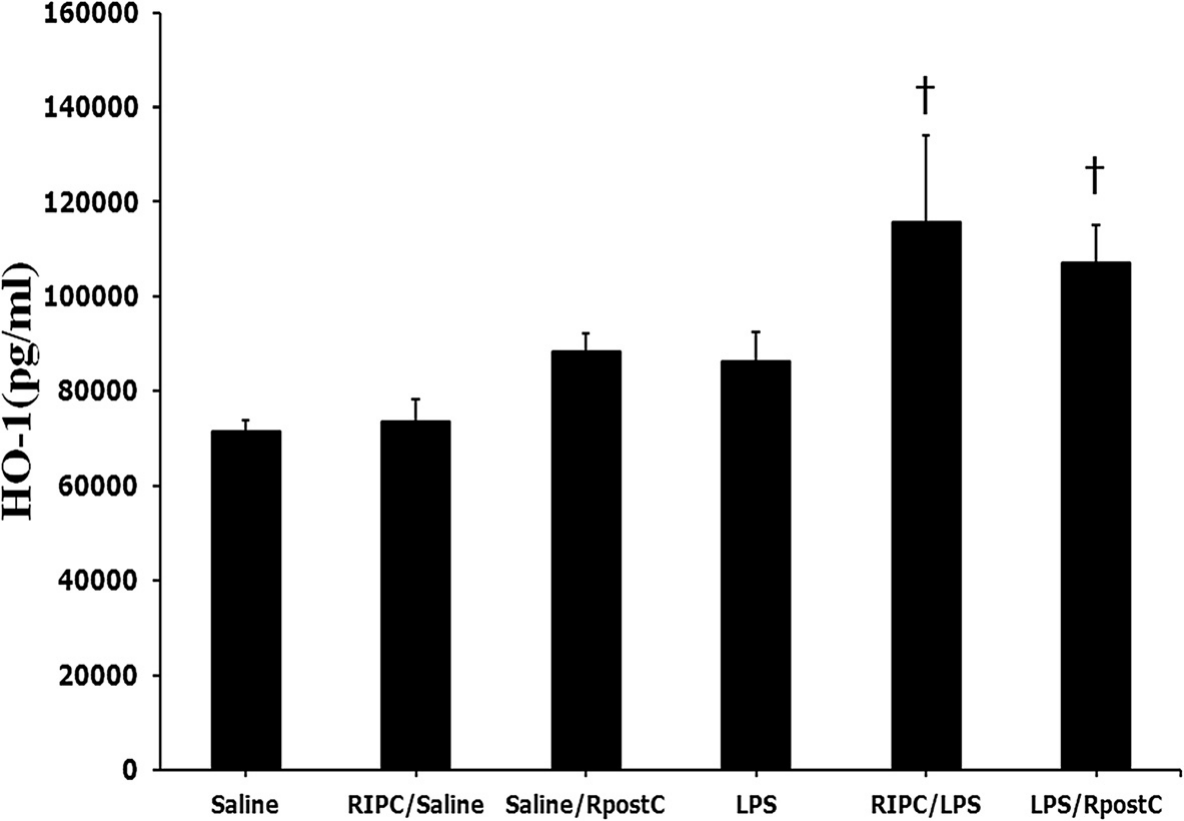
**HO-1 activity.** Quantification of the HO-1 levels was higher in the RIPC/LPS and LPS/RpostC compared with Saline group at 1 h after LPS or saline injection (*p* < 0.05). Mice were treated with saline (Saline group), RIPC plus saline (RIPC/Saline group), saline plus RpostC (Saline/RpostC group), LPS (20 mg/kg, i.p.; LPS group), RIPC plus LPS (RIPC/LPS group), and LPS plus RpostC (LPS/RpostC group). i.p.: intraperitoneal. RIPC: remote ischemic preconditioning, RpostC: remote ischemic postconditioning. Data are expressed as mean ± SEM (n = 6 in each group). *P < 0.05 versus LPS group. † *P* < 0.05 versus Saline.

Quantification of the HO-1 levels was substantially higher in the RIPC/LPS and LPS/RpostC groups compared with the LPS group at 1 hour (Figure 5) but significant difference were not found. There were no significant differences across all groups at 12 hours.

### Liver histopathological changes

**Figure 6:**
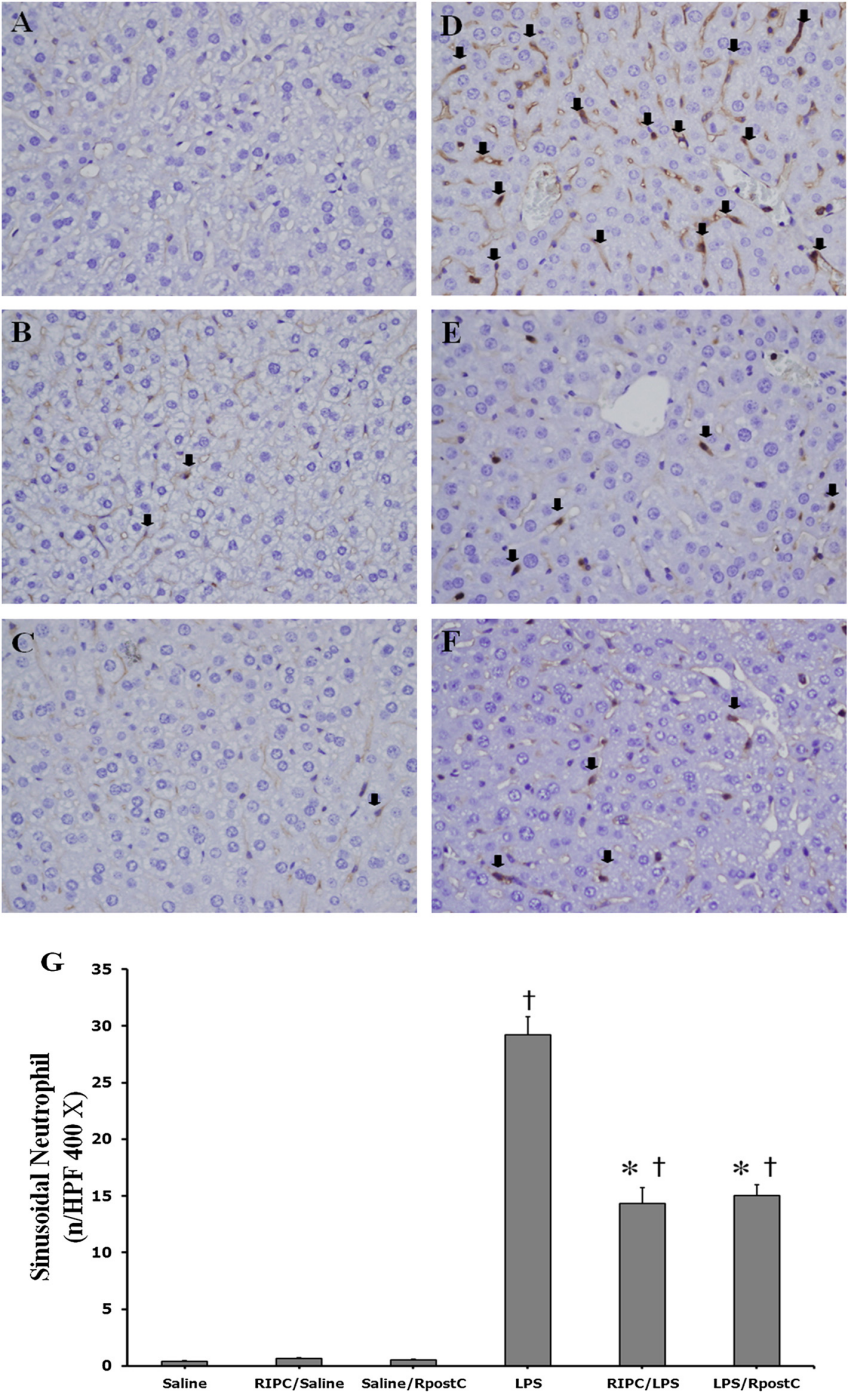
**Liver histopathological changes. (A)** Saline group **(B)** RIPC/Saline **(C)** Saline/RpostC group **(D)** LPS group **(E)** RIPC/LPS group **(F)** LPS/RpostC group. Neutrophil accumulation within liver sinusoid substantially reduced in the RIPC/LPS group **(E)** and LPS/RpostC group **(F)** compared with that in the LPS group **(D)** at 12 h after LPS injection. The brown colored neutrophils are stained with anti-mouse Ly-6G (black arrows). Magnification × 400. **(G)** Quantification of intrahepatic sinusoidal neutrophils by counting PMNs in 16 random high-power fields. Magnification × 400. Mice were treated with saline (Saline group), RIPC plus saline (RIPC/Saline group), saline plus RpostC (Saline/RpostC group), LPS (20 mg/kg, i.p.; LPS group), RIPC plus LPS (RIPC/LPS group), and LPS plus RpostC (LPS/RpostC group). i.p.: intraperitoneal. RIPC: remote ischemic preconditioning, RpostC: remote ischemic postconditioning. Data are expressed as mean ± SEM (n = 6 in each group). *P < 0.05 versus LPS group. Data are † *P* < 0.05 versus Saline, RIPC/Saline, Saline/RpostC.

No significant histologic alterations in the liver specimens were observed in Saline group (Figure 6A), RIPC/Saline (Figure 6B) and Saline/RpostC (Figure 6C). Severe pathologic abnormalities including infiltrating neutrophil cells were seen in the LPS group (Figure 6D). Neutrophil accumulation within the sinusoid of the liver was substantially reduced in the RIPC/LPS (Figure 6E) and LPS/RpostC group (Figure 6F) when compared with that in the LPS group. Quantification of intrahepatic sinusoidal neutrophils by counting the brown color stained PMNs in 16 random high power fields (400×) in the RIPC and RpostC groups revealed a significant neutrophil count decrease compared with that of the LPS group (*p* < 0.001).

## Discussion

RIPC and RpostC improved survival and suppressed pro-inflammatory cytokines, NF-κB activation, and hepatic inflammation in the LPS-induced sepsis mice model. The present study has shown that RpostC has also protective effects on LPS-induced endotoxemia as RIPC.

The survival rate of remote ischemic conditioned mice in the LPS-induced systemic inflammation model amounted to 60-65%, while the survival rate of unconditioned LPS injected mice was 5% in this study. The pharmacologic studies with cerivastatin or imipramine in LPS (15 – 20 mg/kg, i.p.) induced sepsis mice model showed that the survival rate of those treatment groups were 60 – 70% and that survival rate of non-treatment group were 10 – 20% [20,21]. This result suggests that RIPC and RpostC improved survival rates to an extent comparative to some pharmacologic therapies.

Clinical and experimental studies have shown that LPS-induced sepsis can lead to a rapid simultaneous secretion of two functionally heterogeneous groups of cytokines: pro-inflammatory cytokines (e.g., TNF-α, IL-1β and IL-6) and anti-inflammatory (e.g., IL-10) [22]. The correlation between TNF-α or IL-6 level and severity of disease exhibit the importance of these cytokines in septic shock [23-25]. Cytokine levels increased in a dose dependent manner. The dosage of LPS, 20 mg/kg used in this study was chosen as indicated by our preliminary experiment and other studies [26]. A single injection of high-dose LPS induces a very rapid, but transient, systemic cytokines with peak levels between 1.5 and 4 hours that began to decline at 8 hours [27]. Our preliminary study showed that TNF-α peaked at 1 hour after LPS injection, while IL-6 peaked at 4 hours after LPS injection. Therefore, we measured TNF-α, IL-1β, IL-6, and IL-10 plasma at 1 and/or 4 hours after LPS or saline administration.

RIPC significantly suppressed the release of TNF-α and IL-6 and RpostC also significantly suppressed the release of TNF-α, IL-6, and IL-1β increasing the release of anti-inflammatory cytokine IL-10. This was a finding similar to a previous study in which ischemic post-conditioning had caused a significant reduction in systemic inflammatory response [10].

In immune and inflammatory responses, NF-κB is a critical transcription factor and plays a crucial role through the regulation of genes encoding pro-inflammatory cytokines (e.g., TNF-α, IL-1β, IL-6), adhesion molecules, chemokines (e.g., interleukin-8), and monocyte chemotactic protein-1 (MCP-1) [28]. Therefore, NF-κB is activated early in the pathogenesis of organ injury in sepsis [4,5]. In models of sepsis, greater levels of nuclear accumulation of NF-κB are associated with higher rates of mortality and worse clinical outcomes [29]. Contrariwise, suppression of NF-κB activation decreases acute inflammatory responses and organ dysfunction outcome [29] and down-regulation of NF-κB activation could be a suitable therapeutic target in sepsis. This present study provides evidence that RIPC and RpostC decreases NF-κB DNA binding activity in the liver during endotoxemia. Also, this result may be related to in less death in RIPC and RpostC treated groups.

Some studies have reported that NF-κB activation and nuclear translocation are inhibited by HO-1 [6,7]. HO-1 is an enzyme catalyzing the degradation of heme into carbon monoxide, biliverdin, and free iron [11]. HO-1 and its by-products have been shown to promote cytoprotection from oxidative stress, apoptotic cell death, and cell injury during ischemia/reperfusion [30,31]. Ischemic pre-conditioning and post-conditioning protect against I/R injury and reduce systemic pro-inflammatory cytokine release by induction of HO-1 [9-11,30]. More importantly, HO-1 induction by chemical inducers could provide protection against LPS-induced liver injury in rats [19]. HO-1 may diminish extravascular edema formation and endothelial cell swelling by virtue of its anti-inflammatory, anti-apoptotic and anti-proliferative actions, resulting in reduced microvascular compression and improved organ perfusion [32,33]. Another aim of this study was to determine if RpostC are associated with the activation of HO-1 as the protective mechanism for LPS induced hepatic damage. HO-1 activation was substantially increased in the RIPC/LPS and LPS/RpostC groups when compared with that in the LPS group. However, this study did not show significant differences. A previous study showed that intestinal ischemic preconditioning significantly reduced the LPS-induced expression of inflammatory cytokine in intestine and lung, while ischemic preconditioning selectively increased HO-1 mRNA expression in only intestine and there was no expression in lungs (18). Therefore, it is possible that HO-1 activation may not be shown in liver. Further study is need for this HO-1 activation.

Hepatocyte vacuolation and nodular necrosis were observable at 12 hours post-LPS-injection (20 mg/kg, i.p.) and mice began to die within 16 hours of LPS injection in our study [34]. Therefore, liver tissues were collected at 12 hours after LPS injection. Histological examinations of liver sections from LPS-treated mice revealed infiltrated neutrophil and necrotic hepatocytes. However, RIPC and RpostC suppressed significantly LPS-induced intrahepatic sinusoidal neutrophils infiltration. These results seem to be correlated to survival rate.

The anti-inflammatory effects of remote ischemic conditioning were observed in mice, which will most likely yield different results in humans [35] and the effects of RIPC or RpostC could be questionable in septic patients. Nevertheless, intermittent pneumatic compression is routinely applied to ICU patients and it might be used as an alternative method of IPC. Therefore, clinical trials would be needed to establish its potential role for treatment of sepsis in humans.

Severe sepsis and septic shock have been responsible for the high morbidity and mortality among intensive care unit (ICU) patients. Mortality rates have decreased considerably with the discovery of antimicrobial agents. However, morbidity and mortality of sepsis have steadily increased due to the emergence of antimicrobial-resistant microorganisms [36]. Also, antimicrobial agents themselves often result in irreversible organ failure [37]. Therefore, this study attempts to understand innate protective mechanisms and develop new therapies against sepsis. In summary, both RIPC and RpostC attenuated NF-κB activation and reduced production of pro-inflammatory cytokines and increased anti-inflammatory cytokines release and attenuated hepatic injury and improved survival rate in LPS-induced systemic inflammation model. These results suggest that RIPC and RpostC may be a useful therapeutic approach in the treatment of sepsis.

## Conclusions

The present study demonstrated that not only RIPC but RpostC attenuated inflammatory responses and improved survival of mice with LPS-induced systemic inflammation by modifying NF-κB mediated expression of cytokines.

## Abbreviations

ELISA: Enzyme linked immunosorbent assay; HO-1: Heme oxygenase-1; ICU: Intensive care unit; IL-1β: Interleukin-1 beta; IL-6: Interleukin-6; IL-10: Interleukin-10; i.p.: intraperitoneally; I/R: Ischemia and reperfusion; LPS: Lipopolysaccharide; MCP-1: Monocyte chemotactic protein-1; NF-κB: Nuclear factor kappa B; PBS: Phosphate buffered saline; PMNs: Polymorphonuclear neutrophils; RIPC: Remote ischemic preconditioning; RpostC: Remote ischemic postconditioning; SEM: Standard error of the mean; TNF-α: Tumor necrosis factor-alpha.

## Competing interests

The authors declare that they have no competing interests.

## Authors’ contributions

YHK participated in the design of the study, performed several experiments, analysed data and statistics, and wrote the manuscript. DWY performed the experiments, supervised samples analyses. JHK participated in the design, provided clinical expertise and helpful discussions. JHL provided helpful discussions and edited the manuscript. CHL conceived the study, participated in the design of the study, analysed data and statistics, wrote the grant application, and supervised the preparation of the manuscript. All the experiments were performed in Korea University Medical Center’s laboratory. All authors read and approved the final manuscript.
